# Emotional and behavioral features associated with subclinical hypothyroidism in children and adolescents with Down syndrome

**DOI:** 10.3389/fpsyg.2023.1294908

**Published:** 2024-02-06

**Authors:** Elisa Fucà, Floriana Costanzo, Paolo Galassi, Laura Celestini, Alberto Villani, Diletta Valentini, Stefano Vicari

**Affiliations:** ^1^Child and Adolescent Neuropsychiatry Unit, Bambino Gesù Children’s Hospital, IRCCS, Rome, Italy; ^2^Pediatric Unit, Pediatric Emergency Department, Bambino Gesù Children's Hospital, IRCCS, Rome, Italy; ^3^Department of Life Science and Public Health, Catholic University of the Sacred Heart, Rome, Italy

**Keywords:** thyroid dysfunction, trisomy 21, CBCL, parasomnias, sleep–wake transition disorders, psychopathology

## Abstract

**Background:**

Subclinical hypothyroidism (SH) is particularly frequent in individuals with Down syndrome (DS). Despite the amount of evidence suggesting SH is associated with psychopathological symptoms and sleep problems in general population, poor is known about the emotional and behavioral features associated with SH in children with DS.

**Objective:**

The first aim of the current study was to investigate differences in emotional and behavioral profiles between a group of children with DS exhibiting co-occurring SH and a group of age and BMI-matched children with DS without co-occurring SH. The second aim of the present study was to investigate differences in sleep disturbances between these groups.

**Methods:**

We included in this retrospective study 98 participants with DS aged 3–18 years with the aim to explore differences in emotional/behavioral problems as well as in sleep difficulties between children with DS with or without co-occurring SH.

**Results:**

Participants with co-occurring SH exhibited significantly higher scores at several scales of the Conners’ Parent Rating Scales Long Version – Revised. However, they did not exhibit more sleep problems than control group.

**Conclusion:**

These results provide specific indications for psychological and neuropsychiatric evaluation of children with DS with suspected or diagnosed SH, highlighting the importance of a multidisciplinary approach in clinical care for children and adolescents with DS.

## 1 Introduction

Down syndrome (DS) is the most frequent chromosomal condition, affecting 1 in every 787 live born babies (de Graaf et al., 2015, 2017) and the most common genetic cause of intellectual disability. DS is associated with increased risk of several medical issues, such as congenital heart disease, obstructive sleep apnoea, celiac disease, and endocrine abnormalities, including thyroid dysfunctions (Antonarakis et al., 2020). Individuals with DS exhibit a range of thyroid dysfunctions that encompass congenital hypothyroidism, subclinical hypothyroidism (SH), acquired hypothyroidism (autoimmune/non autoimmune), and hyperthyroidism (Pierce et al., 2017; Valentini et al., 2021). For example, higher rates of hypothyroidism have been documented, with estimated prevalence of 23.5% in pediatric age and 39–61% in adult age (Carroll et al., 2008; Tsou et al., 2020). Another thyroid dysfunction particularly frequent in DS is SH, whose prevalence in this population has been estimated up to 87% (O’Grady and Cody, 2011; Szeliga et al., 2022), remarkably higher than the prevalence reported in general population (5%) (Chiovato et al., 2019). SH diagnosis is possible when serum thyroid-stimulating hormone (TSH) is slightly elevated and the levels of total or free thyroxine and triiodothyronine are normal (O’Grady and Cody, 2011). It has been proposed that the increased prevalence of thyroid dysfunctions in youth with DS could be determined by a delayed maturation of the hypothalamic–pituitary-thyroid axis; this would result in a temporary process not necessitating treatment (Sharav, 1988, 1991). Accordingly, literature suggests that only a limited percentage of individuals with DS and SH develops an overt hypothyroidism, particularly females with mostly positive titer of antithyroid autoantibodies (Szeliga et al., 2022).

Research on general population suggests SH is associated with increased prevalence of psychopathological symptoms. In particular, individuals with SH tend to exhibit significantly higher levels of depressive symptoms than individuals without SH (Gulseren et al., 2006; Almeida et al., 2007; Chueire et al., 2007; Demartini et al., 2014). An association between SH and depression symptoms has also been reported in animal models of SH (Ge et al., 2014). Moreover, some authors found that depression symptoms are observed more frequently among individuals with SH than those with overt hypothyroidism (Chueire et al., 2007); however, these results were not confirmed by a recent meta-analysis reporting a stronger association between depression and overt hypothyroidism than with SH (Bode et al., 2021). Literature also suggests associations between SH and anxiety symptoms. A systematic review on the topic highlighted that individuals with an anxiety disorder seem to be significantly more likely to have a co-occurring thyroid disorder (Fischer and Ehlert, 2018). Intriguingly, a recent study explored the relationship between SH and anxiety symptom in young first-episode and drug-naïve patients with major depressive disorder (Wu et al., 2023). The authors reported that high serum TSH level was associated with anxious major depressive disorder in young first-episode and drug-naïve individuals with major depressive disorder and SH, thus suggesting serum TSH level could be a possible biomarker for predicting anxiety symptoms, at least for the population considered in the study.

It has been also suggested that SH could be associated with sleep difficulties. Indeed, individuals with SH seem to exhibit poorer sleep quality in comparison with individuals without thyroid dysfunction (Song et al., 2019; Wu et al., 2021). Results from these studies were in contrast to previous reports suggesting no relationship between SH and sleep quality (Akatsu et al., 2014). Another research, involving 4,945 participants older than 19 years of age, reported significant associations between SH and sleep duration (Kim et al., 2019). Moreover, there are reports of higher frequency of SH among individuals with restless legs syndrome (Geng et al., 2022). On the other hand, contrasting findings have been reported about the existence of relationships between obstructive sleep apnoea and SH (Resta et al., 2005; Ozcan et al., 2014; Petrone et al., 2016; Zhang et al., 2016). As concerns population with DS, thyroid dysfunctions have been associated with behavioral sleep disturbances in adults, such as early awakenings (Esbensen, 2016).

Despite the well-described high prevalence of SH in individuals with DS, poor is known about possible, specific emotional and behavioral correlates of SH in such population, as also underlined by a recent review (Hirsch and Gaultney, 2023). Therefore, the first aim of the current study was to investigate differences in emotional and behavioral profiles between a group of children with DS exhibiting co-occurring SH and a group of age and body mass index (BMI)-matched children with DS without co-occurring SH. The second aim of the present study was to investigate differences in sleep disturbances between these groups. Based on literature, we hypothesized that participants with co-occurring SH would exhibit more internalizing symptoms (i.e., anxiety and depression symptoms) as well as more sleep difficulties than participants without SH did, in particular for insomnia-related difficulties.

## 2 Materials and methods

### 2.1 Procedure

This was a cross-sectional, retrospective study. Data were collected by reviewing electronic health records of 200 children and adolescents with DS referred for a clinical evaluation at the DS center of the Pediatric Department and/or at the Child and Adolescent Neuropsychiatry Unit of the Bambino Gesù Children’s Hospital in Rome between September 2022 and May 2023. At the DS center, participants underwent a clinical assessment consisting in a multidisciplinary evaluation that included visit with a physician with a specific education in DS and with other professionals, e.g., nutritionists, audiologist, endocrinologist. Here, in accordance with recent indication for health supervision for children and adolescents with DS, participants had blood drawn to screen for possible medical comorbidities, including thyroid dysfunction (Bull et al., 2022). The clinical assessment at the Child and Adolescent Neuropsychiatry Unit included a neuropsychiatric, neuropsychological and psychopathological/behavioral assessment by trained clinicians specialized in neuropsychiatric and psychological assessment of children and adolescents with DS. All parents signed a written informed consent for data use for research purposes and a privacy statement that ensures that data will be kept confidential. The study was conducted according to the guidelines of the Declaration of Helsinki and was approved by the local Ethics Committee (process number HGP-T21).

Two trained clinicians extracted the data. The clinicians reached an agreement on which variables to extract and how the coding would be performed before the data extraction occurred. They coded the data independently. The agreement between physicians reached 95%. Discrepancies were discussed, and mutual agreement was achieved.

### 2.2 Participants

Selection criteria included, besides the diagnosis of DS based on the analysis of the karyotype, the age ranging between 3 and 18 years, a SH diagnosis based on thyroid function testing (thyroid blood tests) in children with no evident clinical signs of hypothyroidism, exhibiting TSH levels elevated above 4.0 mIU/ml and thyroxine and triiodothyronine within the reference range. Exclusion criteria were as follows: age < 3 and > 18 years; language barrier hampering questionnaire compilation by parents; diagnosis of autoimmune thyroiditis; ascertained ongoing pharmacological treatment for thyroid dysfunction such as levothyroxine. The final sample included 98 individuals with DS ranging in age from 3 to 18 years of age: 42 with co-occurring SH (SH group - 23 boys, 19 girls; mean age: 9.95 ± 4 years; IQ 53.83 ± 12.1) and 56 age, IQ and sex-matched controls without SH (CON group - 36 boys, 20 girls; mean age: 10.99 ± 3.35 years; IQ 55.53 ± 7.93). Since one of the aims of the current work was to investigate potential differences in sleep difficulties, and considering that risk factors for developing obstructive sleep apnoea and hypothyroidism can both include obesity (Frey and Pilcher, 2003; Sanyal and Raychaudhuri, 2016; Zhang et al., 2016), we matched SH group and CON group also for BMI (21.29 and 20.81, respectively; *p* = 0.656).

### 2.3 Measures

#### 2.3.1 Emotional and behavioral profile

Child Behavior Checklist (CBCL). Emotional and behavioral problems were evaluated by means of the CBCL (Achenbach, 2011). For pre-schoolers, we used the CBCL for ages 1.5 to 5, which consists of 100 problem items. The instrument generates seven syndrome scales and five DSM-oriented scale profiles, consistent with the diagnostic categories of DSM-IV-TR and DSM-5. For participants aged 6–18 years, we used the CBCL 6–18, which generates eight syndrome scales and six DSM-Oriented scales. Both versions (i.e., versions for ages 1.5–5 and 6–18 years) include three general domain scales, namely Internalizing, Externalizing and Total problems. In the current study, we considered the Internalizing and Externalizing problems scales.

Conners’ Parent Rating Scales Long Version – Revised (CPRS). The CPRS (Conners et al., 1998) is a tool for the screening of attention deficit hyperactivity/disorder (ADHD) and related symptoms. The instrument includes 80 items and it is composed of 14 different scales, namely: oppositional; inattention; hyperactivity; anxiety; perfectionism; social problems; psychosomatic problems; ADHD index; Clinical Global Impression Scale (CGI): restlessness; CGI: emotional instability; CGI: total; DSM-IV: inattention; DSM-IV: hyperactivity/impulsivity; DSM-IV: Total. The instrument generates a T-score for each subscale. The cut-off for T-scores for clinical significance is >70 (very elevated) and T-scores from 60 to 70 are considered as high averages or elevated. Data from CPRS were not available for 12 out of 42 participants in the SH group. In the current study, we considered the following scales: ADHD index; CGI: restlessness; CGI: emotional instability; CGI: total; DSM-IV: inattention; DSM-IV: hyperactivity/impulsivity; DSM-IV: Total.

#### 2.3.2 Sleep disturbances

Sleep disturbances were assessed by means of Sleep Disturbance Scale for Children (SDSC) (Bruni et al., 1996), which explores the presence of sleep disorders during the previous 6 months and contains 26 items with Likert scale values of 1–5. The items are subdivided into six sleep disorder subscales: disorders in initiating and maintaining sleep (DIMS), sleep breathing disorders (SBD), disorders of arousal (DA), sleep–wake transition disorders (SWTD), disorders of excessive somnolence (DOES), and sleep hyperhidrosis (SHY).

### 2.4 Statistical analyzes

Descriptive statistics were used to analyze demographic and clinical characteristics of the whole sample. Chi-squared test was used to determine the non-parametric variables. Group differences were examined by t test and analysis of co-variance (ANCOVA); we selected age and sex as covariates considering their potential influence on psychopathological symptoms in DS (Van Gameren-Oosterom et al., 2013; Grieco et al., 2015) and BMI considering that risk factors for developing hypothyroidism can include obesity (Sanyal and Raychaudhuri, 2016). A *p*-value ≤0.05 was considered as statistically significant. To correct for multiple testing, *p*-values were adjusted according to the Benjamini and Hochberg procedure.

## 3 Results

### 3.1 Differences in emotional and behavioral problems

ANCOVAs failed to detect any significant difference between SH group and controls on internalizing nor externalizing problems scores of the CBCL before and after adjusting for multiple correction (all *p* > 0.05). These two broadband scales combine several of the syndrome scales. In particular, the Internalizing problems scale sums the Anxious/depressed, Withdrawn-depressed, and Somatic complaints scores. The lack of group differences on this scale suggests that children with DS and co-occurring SH do not exhibit more anxious and depressive symptoms than participants in the CON group. On the other hand, the Externalizing problems scale combines Rule-breaking and Aggressive behavior. The lack of group differences on this scale suggests that children with DS and co-occurring SH do not exhibit more behavioral problems associated with physical or verbal aggression, hyperactivity, opposition, defiance/disobedience than participants in the CON group. As concerns CPRS, significant differences were observed between the SH group and controls for most of the scales considered. In particular, participants in the SH group exhibited significantly higher scores (suggesting more severe symptomatology) at the following scales: ADHD index (risk for ADHD); CGI: restlessness (restless, impulsive, inattentive); CGI: emotional instability (emotional, cry a lot, get angry easily); CGI: total (hyperactive, broad range behavioral problems) Table 1 summarizes results.

**Table 1:** Differences between CON and CD groups at CBCL and CPRS scores.

	CON	SH	Adjusted *p* values
CBCL			
Internalizing problems	54.07 ± 8.96	56 ± 9.3	0.134
Externalizing problems	53.4 ± 7.7	55.56 ± 8.22	0.19
CPRS			
ADHD index	59.57 ± 11.34	66.43 ± 17	0.05*
CGI: restlessness	56.37 ± 10.93	63.6 ± 16.04	0.049*
CGI: emotional instability	50.91 ± 14.79	60.47 ± 12.88	0.014*
CGI: total	55.11 ± 10.96	63.3 ± 16.64	0.031*
DSM-IV: inattention	58.93 ± 12.52	65 ± 14.47	0.06
DSM-IV: hyperactivity/impulsivity	53.84 ± 13.06	58.13 ± 14.79	0.115
DSM-IV: Total	56.62 ± 11.95	62.43 ± 14.81	0.06

*p ≤ 0.05.

### 3.2 Differences in sleep disturbances

ANCOVAs failed to detect significant group differences at the SDSC scales. Indeed, after implementing multiple testing correction, adjusted *p*-values were all >0.05, indicating that participants in the SH group did not show higher scores on any of the questionnaire scales. This suggests that children with DS and co-occurring SH do not exhibit more sleep disturbances associated with difficulties in falling asleep, night-time awakenings, sleep-related breathing disorders, parasomnias, excessive somnolence nor sleep hyperhidrosis than participants in the CON group. Table 2 summarizes results.

**Table 2:** Differences between CON and SH groups in SDSC scores.

	CON	SH	Adjusted *p* values
DIMS	56.41 ± 13.06	61.9 ± 15.32	0.188
SBD	60.78 ± 15.53	61.02 ± 16.06	0.823
DA	58.62 ± 14.98	57.67 ± 14.92	0.701
SWTD	56.28 ± 15.11	63.46 ± 17.5	0.1
DOES	50.55 ± 9.37	53.48 ± 12	0.198
SHY	49.73 ± 7.34	53.71 ± 13.31	0.15

DIMS, disorders in initiating and maintaining sleep; SBD, sleep breathing disorders; DA, disorders of arousal; SWTD, sleep–wake transition disorders; DOES, disorders of excessive somnolence; SHY, sleep hyperhidrosis.

## 4 Discussion

The first aim of the present work was to investigate differences in emotional and behavioral problems between a group of children with DS exhibiting co-occurring SH and a group of children with DS without co-occurring SH. Contrary to our hypothesis, we did not observe any group difference on internalizing problems as detected by the CBCL. Instead, participants in the SH group exhibited higher scores in several scales indicating externalizing symptomatology, as detected by the CPRS, namely: ADHD index; CGI: restlessness; CGI: emotional instability; CGI: total. These findings then suggest that, in children and adolescents with DS, the presence of SH is associated with externalizing rather than internalizing problems, taking into account confounding factors such as age, BMI or sex. Although most of the available evidence in typical population points to an association between SH and internalizing problems, it is possible to identify some indications of externalizing symptomatology in association with hypothyroid conditions. For instance, there are indications for a higher prevalence of SH in children and adolescents with behavioral dysregulation (Holtmann et al., 2010). Another study conducted on a sample of 224 children with SH aged 11–19 years reported impaired emotional intelligence quotients, including worse self-control than controls (Arianas et al., 2022). Intriguingly, these studies focused exclusively on pediatric age, similar to the present research. Preclinical studies also suggest that juvenile hypothyroidism is associated with hyperactive locomotor behavioral patterns (Van Wijk et al., 2008; Umezu et al., 2019). Our findings provide preliminary evidence for considering SH-related problems in children with DS who exhibit behavioral changes associated with hyperactivity.

The second aim of the current study was to investigate differences in sleep problems between children with and without co-occurring SH. We found that participants in the SH group did not exhibit significantly higher scores at the SDSC compared to participants in the control group. This finding is inconsistent with some studies that suggest an association between SH and sleep difficulties (Iodice et al., 2019; Kim et al., 2019; Song et al., 2019; Green et al., 2021; Geng et al., 2022). Such inconsistency could be explained, at least in part, by differences in the method used to detect the presence of sleep disorders: some studies used self-report questionnaires specifically designed for the research, others used standardized questionnaires, and still others employed objective measures, such as polysomnography. However, the association between SH and sleep disturbances has not been unequivocally reported in the literature. For instance, the study by Gencer et al. (2022) included 120 participants, of whom 60 had newly diagnosed SH and 60 had normal thyroid functions; the authors did not detect any significant difference in the frequency and severity of obstructive sleep apneas measured by polysomnography (Gencer et al., 2022). The absence of group differences in an indirect index of sleep-related breathing disorders, as provided by SBD scale scores of the SDSC, could be explained by two reasons. The first one relates with the general vulnerability of individuals with DS to sleep-related breathing difficulties, such as sleep apneas (Hirsch and Gaultney, 2023), as also confirmed by the mean scores of the SBD scale that fall into borderline range for both groups. Moreover, the BMI matching between SH and CON groups could contribute in explaining the lack of group differences at SBD scores, since obesity could be a confounding factor in the association between SH and sleep apneas, as previously suggested (Zhang et al., 2016). However, participants included in the current research did not undergo polysomnography; we only collected parent-reported information on child’s sleep. Therefore, the comparison between the results emerging from the present study and those reported in the literature is limited by methodological differences.

This study is not without limitations. The first one is the restricted sample size, which limits the generalizability of the results. The second limitation is the wide age range of the participants included: since the risk of hypothyroidism increases with age in individuals with DS (Bull et al., 2022), future research should focus on specific age ranges in order to better characterize emotional and behavioral features associated with SH across development. The use of parent-report questionnaires to evaluate emotional and behavioral problems, as well as sleep difficulties, represents another limitation of this study. Future studies should include additional tools for psychological assessment, such as clinical interviews, and utilize objective measures to detect sleep disturbances, such as polysomnography and actigraphy.

Despite these limitations, the present research provides evidence of specific emotional and behavioral features associated with SH in children and adolescents with DS. Recently developed guidelines suggest evaluating for medical problems that can be associated with behavior changes, including thyroid abnormalities, for children with DS aged 5–12 years (Bull et al., 2022). This study has useful clinical implications, given that it offers specific indications for clinicians involved in the follow-up of children with DS about the need to pay attention to the development of behavioral problems in children with DS and co-occurring SH. A multidisciplinary approach that includes diverse professionals, such as pediatricians, psychologists, neuropsychiatrists, and endocrinologists, is essential to for the healthcare of children and adolescents with DS.

## Data availability statement

The raw data supporting the conclusions of this article will be made available by the authors, without undue reservation.

## Ethics statement

The studies involving humans were approved by Ospedale Pediatrico Bambino Gesù. The studies were conducted in accordance with the local legislation and institutional requirements. Written informed consent for participation in this study was provided by the participants' legal guardians/next of kin.

## Author contributions

EF: Conceptualization, Data curation, Formal analysis, Investigation, Methodology, Writing – original draft, Writing – review & editing. FC: Conceptualization, Formal analysis, Investigation, Methodology, Writing – original draft, Writing – review & editing. PG: Data curation, Investigation, Writing – original draft. LC: Data curation, Writing – original draft. AV: Writing – review & editing. DV: Conceptualization, Writing – review & editing. SV: Conceptualization, Project administration, Supervision, Writing – review & editing.

## References

[ref1] Achenbach T. M. (2011). “Child behavior checklist” in Encyclopedia of clinical neuropsychology. eds. Kreutzer J. S. DeLuca J. Caplan B. (New York, NY: Springer New York), 546–552.

[ref2] Akatsu H. Ewing S. K. Stefanick M. L. Fink H. A. Stone K. L. Barrett-Connor E.. (2014). Association between thyroid function and objective and subjective sleep quality in older men: the osteoporotic fractures in men (MrOS) study. Endocr. Pract. 20, 576–586. doi: 10.4158/EP13282.OR, PMID: 24449663 PMC4363141

[ref3] Almeida C. Brasil M. A. Costa A. J. L. Reis F. A. A. Reuters V. Teixeira P.. (2007). Subclinical hypothyroidism: psychiatric disorders and symptoms. Rev. Bras. Psiquiatr. 29, 157–159. doi: 10.1590/S1516-44462007000200013 17639255

[ref4] Antonarakis S. E. Skotko B. G. Rafii M. S. Strydom A. Pape S. E. Bianchi D. W.. (2020). Down syndrome, Down syndrome. Nat. Rev. Dis. Primers. 6:9. doi: 10.1038/s41572-019-0143-7, PMID: PMC8428796 32029743

[ref5] Arianas G. Κ. Kostopoulou E. Ioannidis A. Dimopoulos I. Chiotis C. Prezerakos P.. (2022). Emotional intelligence scores in children and adolescents with subclinical hypothyroidism—correlation with serum serotonin and thyroid-stimulating hormone (TSH) concentrations. Hormones 21, 53–60. doi: 10.1007/s42000-021-00320-3, PMID: 34780029

[ref6] Bode H. Ivens B. Bschor T. Schwarzer G. Henssler J. Baethge C. (2021). Association of Hypothyroidism and Clinical Depression: a systematic review and Meta-analysis. JAMA Psychiatry 78, 1375–1383. doi: 10.1001/jamapsychiatry.2021.2506, PMID: 34524390 PMC8444067

[ref7] Bruni O. Ottaviano S. Guidetti V. Romoli M. Innocenzi M. Cortesi F.. (1996). The sleep disturbance scale for children (SDSC) construct ion and validation of an instrument to evaluate sleep disturbances in childhood and adolescence. J. Sleep Res. 5, 251–261. doi: 10.1111/j.1365-2869.1996.00251.x, PMID: 9065877

[ref8] Bull M. J. Trotter T. Santoro S. L. Christensen C. Grout R. W. THE COUNCIL ON GENETICS (2022). Health supervision for children and adolescents with down syndrome. Pediatrics 149:e2022057010. doi: 10.1542/peds.2022-057010, PMID: 35490285

[ref9] Carroll K. N. Arbogast P. G. Dudley J. A. Cooper W. O. (2008). Increase in incidence of medically treated thyroid disease in children with down syndrome after rerelease of American Academy of Pediatrics health supervision guidelines. Pediatrics 122, e493–e498. doi: 10.1542/peds.2007-3252, PMID: 18606626 PMC2666985

[ref10] Chiovato L. Magri F. Carlé A. (2019). Hypothyroidism in context: where We’ve been and where We’re going. Adv. Ther. 36, 47–58. doi: 10.1007/s12325-019-01080-8, PMID: 31485975 PMC6822815

[ref11] Chueire V. B. Romaldini J. H. Ward L. S. (2007). Subclinical hypothyroidism increases the risk for depression in the elderly. Arch. Gerontol. Geriatr. 44, 21–28. doi: 10.1016/j.archger.2006.02.001, PMID: 16678286

[ref12] Conners C. K. Sitarenios G. Parker J. D. A. Epstein J. N. (1998). No title found. J. Abnorm. Child Psychol. 26, 257–268. doi: 10.1023/A:1022602400621 9700518

[ref13] de Graaf G. Buckley F. Skotko B. G. (2015). Estimates of the live births, natural losses, and elective terminations with down syndrome in the United States. Am. J. Med. Genet. 167, 756–767. doi: 10.1002/ajmg.a.37001, PMID: 25822844

[ref14] de Graaf G. Buckley F. Skotko B. G. (2017). Estimation of the number of people with down syndrome in the United States. Genet. Med. 19, 439–447. doi: 10.1038/gim.2016.127, PMID: 27608174

[ref15] Demartini B. Ranieri R. Masu A. Selle V. Scarone S. Gambini O. (2014). Depressive symptoms and major depressive disorder in patients affected by subclinical hypothyroidism: a cross-sectional study. J. Nerv. Ment. Dis. 202, 603–607. doi: 10.1097/NMD.0000000000000168, PMID: 25010109

[ref16] Esbensen A. J. (2016). Sleep problems and associated comorbidities among adults with down syndrome: sleep problems in adults with down syndrome. J. Intellect. Disabil. Res. 60, 68–79. doi: 10.1111/jir.12236, PMID: 26521721 PMC5424472

[ref17] Fischer S. Ehlert U. (2018). Hypothalamic-pituitary-thyroid (HPT) axis functioning in anxiety disorders. A systematic review. Depress. Anxiety 35, 98–110. doi: 10.1002/da.22692, PMID: 29064607

[ref18] Frey W. C. Pilcher J. (2003). Obstructive sleep-related breathing disorders in patients evaluated for bariatric surgery. Obes. Surg. 13, 676–683. doi: 10.1381/096089203322509228, PMID: 14627460

[ref19] Ge J.-F. Peng Y.-Y. Qi C.-C. Chen F.-H. Zhou J.-N. (2014). Depression-like behavior in subclinical hypothyroidism rat induced by hemi-thyroid electrocauterization. Endocrine 45, 430–438. doi: 10.1007/s12020-013-0001-4, PMID: 23794115

[ref20] Gencer A. Atahan E. Kadioglu P. Mutlu B. (2022). Investigation of the frequency of obstructive sleep apnoea syndrome in patients with subclinical hypothyroidism. ERJ Open Res 8, 00186–02022. doi: 10.1183/23120541.00186-2022 36299364 PMC9589324

[ref21] Geng C. Yang Z. Kong X. Xu P. Zhang H. (2022). Association between thyroid function and disease severity in restless legs syndrome. Front. Neurol. 13:974229. doi: 10.3389/fneur.2022.974229, PMID: 36034269 PMC9412235

[ref22] Green M. E. Bernet V. Cheung J. (2021). Thyroid dysfunction and sleep disorders. Front. Endocrinol. 12:725829. doi: 10.3389/fendo.2021.725829, PMID: 34504473 PMC8423342

[ref23] Grieco J. Pulsifer M. Seligsohn K. Skotko B. Schwartz A. (2015). Down syndrome: cognitive and behavioral functioning across the lifespan. American J Med Genetics Pt C 169, 135–149. doi: 10.1002/ajmg.c.31439, PMID: 25989505

[ref24] Gulseren S. Gulseren L. Hekimsoy Z. Cetinay P. Ozen C. Tokatlioglu B. (2006). Depression, anxiety, health-related quality of life, and disability in patients with overt and subclinical thyroid dysfunction. Arch. Med. Res. 37, 133–139. doi: 10.1016/j.arcmed.2005.05.008, PMID: 16314199

[ref25] Hirsch S. Gaultney J. (2023). Sleep disturbances in individuals with down syndrome: an overview. J. Intellect. Disabil. 27:174462952311730. doi: 10.1177/17446295231173011 37105757

[ref26] Holtmann M. Duketis E. Goth K. Poustka L. Boelte S. (2010). Severe affective and behavioral dysregulation in youth is associated with increased serum TSH. J. Affect. Disord. 121, 184–188. doi: 10.1016/j.jad.2009.06.009, PMID: 19564046

[ref27] Iodice A. Carecchio M. Zorzi G. Garavaglia B. Spagnoli C. Salerno G. G.. (2019). Restless legs syndrome in NKX2-1-related chorea: an expansion of the disease spectrum. Brain Dev. 41, 250–256. doi: 10.1016/j.braindev.2018.10.001, PMID: 30352709

[ref28] Kim W. Lee J. Ha J. Jo K. Lim D.-J. Lee J.-M.. (2019). Association between sleep duration and subclinical thyroid dysfunction based on nationally representative data. JCM 8:2010. doi: 10.3390/jcm8112010, PMID: 31752113 PMC6912782

[ref29] O’Grady M. Cody D. (2011). Subclinical hypothyroidism in childhood. Arch. Dis. Child. 96, 280–284. doi: 10.1136/adc.2009.181800 20413616

[ref30] Ozcan K. M. Selcuk A. Ozcan I. Ozdas T. Ozdogan F. Acar M.. (2014). Incidence of hypothyroidism and its correlation with polysomnography findings in obstructive sleep apnea. Eur. Arch. Otorhinolaryngol. 271, 2937–2941. doi: 10.1007/s00405-014-2962-1, PMID: 24609648

[ref31] Petrone A. Mormile F. Bruni G. Quartieri M. Bonsignore M. R. Marrone O. (2016). Abnormal thyroid hormones and non-thyroidal illness syndrome in obstructive sleep apnea, and effects of CPAP treatment. Sleep Med. 23, 21–25. doi: 10.1016/j.sleep.2016.07.002, PMID: 27692273

[ref32] Pierce M. J. LaFranchi S. H. Pinter J. D. (2017). Characterization of thyroid abnormalities in a large cohort of children with down syndrome. Horm. Res. Paediatr. 87, 170–178. doi: 10.1159/000457952, PMID: 28259872 PMC5483988

[ref33] Resta O. Carratù P. Carpagnano G. E. Maniscalco M. Di Gioia G. Lacedonia D.. (2005). Influence of subclinical hypothyroidism and T4 treatment on the prevalence and severity of obstructive sleep apnoea syndrome (OSAS). J. Endocrinol. Investig. 28, 893–899. doi: 10.1007/BF03345320, PMID: 16419491

[ref001] Sanyal D. Raychaudhuri M. (2016). Hypothyroidism and obesity: An intriguing link. Indian J Endocrinol Metab. 20, 554–557. doi: 10.4103/2230-8210.183454, PMID: 27366725 PMC4911848

[ref35] Sharav T. (1988). Growth studies in infants and children with Down’s syndrome and elevated levels of thyrotropin. Arch. Pediatr. Adolesc. Med. 142, 1302–1306. doi: 10.1001/archpedi.1988.02150120056040, PMID: 2973745

[ref36] Sharav T. (1991). Age-related patterns of thyroid-stimulating hormone response to thyrotropin-releasing hormone stimulation in down syndrome. Arch. Pediatr. Adolesc. Med. 145:172. doi: 10.1001/archpedi.1991.02160020064018 1825262

[ref37] Song L. Lei J. Jiang K. Lei Y. Tang Y. Zhu J.. (2019). The association between subclinical hypothyroidism and sleep quality: a population-based study. RMHP 12, 369–374. doi: 10.2147/RMHP.S234552, PMID: 31908553 PMC6927586

[ref38] Szeliga K. Antosz A. Skrzynska K. Kalina-Faska B. Januszek-Trzciakowska A. Gawlik A. (2022). Subclinical hypothyroidism as the Most common thyroid dysfunction status in children with Down’s syndrome. Front. Endocrinol. 12:782865. doi: 10.3389/fendo.2021.782865 PMC8764180 35058880

[ref39] Tsou A. Y. Bulova P. Capone G. Chicoine B. Gelaro B. Harville T. O.. (2020). Medical Care of Adults with down Syndrome: a clinical guideline. JAMA 324:1543. doi: 10.1001/jama.2020.17024 33079159

[ref40] Umezu T. Kita T. Morita M. (2019). Hyperactive behavioral phenotypes and an altered brain monoaminergic state in male offspring mice with perinatal hypothyroidism. Toxicol. Rep. 6, 1031–1039. doi: 10.1016/j.toxrep.2019.10.005, PMID: 31673505 PMC6816216

[ref41] Valentini D. Di Camillo C. Mirante N. Vallogini G. Olivini N. Baban A.. (2021). Medical conditions of children and young people with down syndrome. J. Intellect. Disabil. Res. 65, 199–209. doi: 10.1111/jir.12804 33426738

[ref42] Van Gameren-Oosterom H. B. M. Fekkes M. Van Wouwe J. P. Detmar S. B. Oudesluys-Murphy A. M. Verkerk P. H. (2013). Problem behavior of individuals with down syndrome in a Nationwide cohort assessed in late adolescence. J. Pediatr. 163, 1396–1401. doi: 10.1016/j.jpeds.2013.06.054, PMID: 23916224

[ref43] Van Wijk N. Rijntjes E. Van De Heijning B. J. M. (2008). Perinatal and chronic hypothyroidism impair behavioural development in male and female rats: hypothyroidism and behavioural development. Exp. Physiol. 93, 1199–1209. doi: 10.1113/expphysiol.2008.042416, PMID: 18567604

[ref44] Wu S. Wang H. Zhou Y. Xia X. Yue Y. Wu Y.. (2023). Clinical correlates of autoimmune thyroiditis and non-autoimmune hypothyroidism in treatment-naïve patients with major depressive disorders. J. Affect. Disord. 323, 755–761. doi: 10.1016/j.jad.2022.12.037, PMID: 36529413

[ref45] Wu K. Zhou Y. Ke S. Huang J. Gao X. Li B.. (2021). Lifestyle is associated with thyroid function in subclinical hypothyroidism: a cross-sectional study. BMC Endocr. Disord. 21:112. doi: 10.1186/s12902-021-00772-z, PMID: 34049544 PMC8161919

[ref46] Zhang M. Zhang W. Tan J. Zhao M. Zhang Q. Lei P. (2016). Role of hypothyroidism in obstructive sleep apnea: a meta-analysis. Curr. Med. Res. Opin. 32, 1059–1064. doi: 10.1185/03007995.2016.1157461, PMID: 26907534

